# Immunization with a recombinant subunit OspA vaccine markedly impacts the rate of newly acquired *Borrelia burgdorferi* infections in client-owned dogs living in a coastal community in Maine, USA

**DOI:** 10.1186/s13071-015-0676-x

**Published:** 2015-02-10

**Authors:** Andrew K Eschner, Kristen Mugnai

**Affiliations:** Merial Limited, 3239 Satellite Boulevard NW, Duluth, GA 30096 USA; Coastal Veterinary Care, 161 Gardiner Road, Wiscasset, ME 04578 USA

**Keywords:** *Borrelia burgdorferi*, Canine, Lyme, Borreliosis, Vaccine, Recombinant, OspA, Veterinary, Antibodies

## Abstract

**Background:**

In North America, *Borrelia burgdorferi* is the causative bacterial agent of canine Lyme borreliosis and is transmitted following prolonged attachment and feeding of vector ticks, *Ixodes scapularis* or *Ixodes pacificus*. Its prevention is predicated upon tick-avoidance, effective on-animal tick control and effective immunization strategies. The purpose of this study is to characterize dogs that are newly seropositive for *Borrelia burgdorferi* infection in relation to compliant use of a recombinant OspA canine Lyme borreliosis vaccine. Specifically, Preventive Fractions (PF) and Risk Ratios (RR) associated with the degree of vaccine compliancy (complete versus incomplete) are determined.

**Methods:**

6,202 dogs were tested over a five year period in a single veterinary hospital utilizing a non-adjuvanted, recombinant OspA vaccine according to a 0, 1, 6 month (then yearly) protocol. Rates of newly acquired “Lyme-positive” antibody test results were compared between protocol compliant and poorly compliant (incompletely and/or non-vaccinated) dogs.

**Results:**

Over the five-year span, one percent (range 0.39 - 1.3) of protocol compliant vaccinated, previously antibody negative dogs became seropositive for infection. Approximately twenty-one percent (range 16.8 – 33.3) of incompletely vaccinated dogs became positive for infection-specific antibodies. The Preventative Fraction for testing positive for antibodies specific for infection with *Borrelia burgdorferi* in any given year based on optimal vaccine compliance was, on average, 95.3% (range 93.29 - 98.08). The Risk Ratio for becoming infected with *Borrelia burgdorferi* antibodies in any given year if vaccine non-compliant was 21.41 (range 14.9 – 52.1). There was a high statistically significant relationship (p = <0.0001) in the observed data in terms of vaccination protocol compliance and the probability of *Borrelia burgdorferi* infection in each of the five years under study.

**Conclusions:**

The recombinant outer surface protein A (rOspA) vaccine for dogs is highly effective in preventing new seropositive cases of *Borrelia burgdoferi* infection over a five-year period in dogs living in an endemic area. Dogs that were vaccine protocol-compliant were significantly less likely to become infected (as indirectly assessed by antibody) with the agent of canine Lyme borreliosis as measured by Preventive Fraction and Risk Ratio calculations.

## Background

### Canine lyme borreliosis

In the United States, canine Lyme borreliosis was first described in 1984 [1]. Infection and disease results from the successful transmission of the spirochete, *Borrelia burgdorferi*, from an infected tick to a dog [1]. The ecology, biology and disease manifestations of *Borrelia burgdorferi*, as well as its sister species in Europe (*B. afzellii* and *B. garinii*) and its tick vector in the *Ixodes* genus have been thoroughly described elsewhere in the literature [2-5].

Four brands, representing two distinct classes of vaccines, are available for use in dogs in the United States for the purpose of preventing Lyme borreliosis. To date, five placebo-controlled laboratory studies pertaining to commercialized whole-cell Lyme borreliosis bacterins and recombinant OspA vaccines for dogs have been published [6-10]. In addition, four published papers have explored the performance of canine Lyme disease vaccines in the private, clinical practice setting [11-14]. Testing parameters, including time frames, site locations, and sample size were dissimilar within all of these studies so direct comparison of vaccine efficacy are not feasible. While whole cell bacterins are appreciably efficacious depending on which parameter you are interested in, reports have described the occasional occurrence of post-vaccinal “Lyme-like” clinical signs in some recently vaccinated, uninfected dogs [7,15,16]. These signs have been putatively associated with the wider array of proinflammatory outer surface *Borrelia* proteins contained within bacterins [15,16]. In an effort to decrease the adverse reaction profile and remedy the occasional diagnostic conundrums posed by whole cell bacterins for Lyme borreliosis, a Type I recombinant OspA (rOspA) subunit extract was licensed and marketed for canine use in the United States in 1996. This vaccine was the first Lyme disease vaccine to be licensed by the USDA demonstrating a 1-year duration of immunity (DOI) after use of a natural tick challenge model [9]. Efficacy of the rOspA vaccine was based on the ability of the vaccine to prevent spirochete transmission as assessed by re-isolation of spirochetes from skin biopsies of vaccinated and non-vaccinated control dogs. In 2000, a randomized, placebo-controlled study explored the effectiveness of the recombinant OspA vaccine (Recombitek® Lyme, Merial) and demonstrated that non-adjuvanted recombinant OspA completely prevented *B. burgdorferi* infection in vaccinated dogs [10].

The following analysis offers a comprehensive look at the performance of the recombinant OspA Lyme-disease vaccine over five years of continuous use in a busy, 3-doctor small (companion) animal practice in Wiscasset, Maine. Since 2009, the practice has been vaccinating dogs to prevent canine Lyme borreliosis utilizing the non-adjuvanted, recombinant OspA subunit vaccine (Recombitek® Lyme, Merial) using an alternate dose veterinary protocol first suggested by Töpfer and Straubinger [17]. These authors observed that “*regardless of vaccine used, the third vaccination induced significantly higher antibody levels*.” This protocol modification suggestion was predicated on earlier work in human medicine showing that the addition of a third dose of vaccine within 6 months of starting the basic immunization protocol could increase the proportion of protected individuals by a factor of almost 2-fold [18]. With emphasis on preventing canine Lyme borreliosis utilizing the Töpfer-Straubinger vaccine protocol recommendations, this Maine veterinary practice advanced the initial annual Lyme disease booster vaccine forward in time by six months. The goal was to raise antibody levels to their maximum more quickly within a dog’s first year and thus they adopted the 0, 1, 6 month vaccine protocol instead of the traditional 0, 1, 12 month framework. Accordingly, the veterinary hospital described in this paper chose to include the recombinant OspA vaccine as a *CORE* vaccine in their vaccine repertoire beginning at 9 weeks of age, utilize a modified protocol and to booster yearly in order to minimize the rate of new *B. burgdorferi* infections in their canine patients. Diagnostic assessment of Lyme ‘positive’ or ‘negative’ was made using a popular test kit (SNAP®) manufactured by IDEXX Laboratories. SNAP® is a proprietary rapid, patient-side, lateral flow ELISA technology platform that can detect antibody or antigen to a variety of vector-borne parasites including C6 antibody to *Borrelia burgdorferi,* antibody to *Ehrlichia canis*, and antigen of the canine heartworm *Dirofilaria immitis* (SNAP® 3Dx). Currently, antibodies to *Anaplasma phagocytophilum* and *Ehrlichia ewingii* can also be detected (SNAP® 4Dx Plus). Further information pertaining to product validation, testing and sensitivity/specificity can be read elsewhere [19].

## Methods

From 2009–2010, the hospital utilized SNAP 3Dx; from 2011–2012, SNAP 4Dx; and for 2013, SNAP 4Dx Plus; the change in kit-type usage over time was the result of increased pathogen testing capabilities by the kit itself and each successive new product was incorporated soon after market entry. The analysis of new Lyme infection rates in vaccinated versus incompletely vaccinated dogs was conducted annually at the conclusion of each year beginning in 2009 until the end of 2013. Utilizing the hospital’s veterinary management software (AVImark®, Piedmont, MO), the results of all SNAP tests conducted in dogs during the analysis year were tallied. Dogs that tested SNAP positive for infection-specific *B. burgdorferi*-antibody were characterized according to vaccine status and ‘age’ of their positive-status. For the analysis, the following parameters/definitions were set. **TEST “AGE”:** A ‘***NEW***’ SNAP *B. burgdorferi*-infection positive result (‘blue-dot’) was defined as a positive-test on a dog that had tested negative on its *most* previously recorded test. An ‘***OLD***’ SNAP *Bb* infection-positive was defined as a repeat positive-test in a dog that *also* tested positive on its most recent previous test. For purposes of this retrospective study, dogs labelled as ‘OLD-positive’ were *not* counted in the analysis of new infection rates in the universe of vaccinated or non-vaccinated dogs within that year. **VACCINE STATUS:** A ‘***FULLY***‘ vaccinated dog was defined as a dog that had adhered to the hospital vaccine recommendation for interval and compliance for at least one year following a negative SNAP test for *B. burgdorferi* antibodies. An ‘***INCOMPLETELY*** vaccinated’ dog was segmented into the degree of incompleteness. ‘***PARTIALLY*** vaccinated’ was defined as having received an initial vaccine with no boosters or having completed its first series of two immunizations with no subsequent booster. ‘***NON-VACCINATED****’* was defined as having had no vaccines in its life or being greater than 15 months *beyond* its regularly scheduled yearly booster.

### Analysis of new-positive infection rates

New SNAP *B. burgdorferi*-infection positive test results were sorted based on the occurrence in the ‘Fully’ vaccinated, protocol-compliant group and the ‘incompletely’ vaccinated/non-vaccinated dogs. For the purpose of this study, **Infection rate (IR)** differences were determined by examining the proportion of *new*-positive test results in fully vaccinated dogs amongst all fully vaccinated dogs tested that year. Likewise, the number of new-positive test results in incompletely vaccinated dogs (either partial or non-vaccinated) was compared to the total of *all* incompletely vaccinated dogs.

**Risk Ratio(RR),** the ratio of the probability of an event (for example, developing a positive test) occurring in an incompletely vaccinated group to the probability of the event occurring in a comparison group (Fully-vaccinated group), were calculated for each year 2009–2013 according to the following equation [20].

$$ \mathbf{Risk}\ \mathbf{Ratio} = \left(\mathbf{C}{\mathbf{I}}_{\mathbf{u}}\right)\ /\ \left(\mathbf{C}{\mathbf{I}}_{\mathbf{e}}\right) $$

[where CI_u_ is the cumulative incidence in the group ‘unexposed’ to Full vaccination and CI_e_ is the cumulative incidence in the group ‘exposed’ to Full vaccination].

**Preventive Fraction (PF) for Benefit of Vaccination**, the percentage of infection reduction in the group exposed to Full vaccination that can actually be attributed to the ‘benefit of full vaccine exposure’, was calculated each year by dividing the risk reduction between two groups (incidence in un*exposed (incompletely vaccinated)* minus incidence in exposed (Fully vaccinated) group by the incidence in the unexposed (expressed as a percent) according to the following equation [20].

$$ \mathbf{P}\mathbf{reventive}\ \mathbf{F}\mathbf{raction} = \mathbf{P}\mathbf{F} = \left[\left(\mathbf{C}{\mathbf{I}}_{\mathbf{u}}\right) - \left(\mathbf{C}{\mathbf{I}}_{\mathbf{e}}\right)\right]\ /\ \left(\mathbf{C}{\mathbf{I}}_{\mathbf{u}}\right)\ \mathbf{x}\ \mathbf{100} $$

[where CI_u_ is the cumulative incidence in the group ‘unexposed’ to Full vaccination and CI_e_ is the cumulative incidence in the group ‘exposed’ to Full vaccination].

## Results

During the five years of analysis (2009–2013), 6,202 SNAP® tests were completed on 4,551 well vaccinated and 1,651 incompletely vaccinated dogs that had previously been characterized as either SNAP ‘positive’ or ‘negative’ (prior to study onset). In total, 835 tests (13.5%) were positive indicating the presence of C6 antibodies specific for infection with *Borrelia burgdorferi*. Inspection of patient records revealed that 432 of the positive tests were in dogs that had also tested positive on their most recent previous test, therefore labeled as ‘*OLD’* positives, and were excluded from the exercise of determining the rate of new infections in vaccinated dogs. Four hundred three dogs were *NEW* positives in the year tested (had tested negative on their most recent previous test), and made up 6.5% of all SNAP tests performed. In the population of 6,202 dogs tested during 2009–2013, 4551 were *fully* vaccinated against canine Lyme borreliosis and compliant with hospital vaccine-protocol recommendations [Table 1]. There were 46 new positive tests among these *fully* vaccinated dogs. One thousand six hundred fifty one tests occurred in *incompletely* vaccinated dogs and among these, 357 were newly positive.

**Table 1:** **Tabulation of yearly testing results for antibodies directed against C6 antigen of** ***Borrelia burgdorferi*** **in dogs utilizing the SNAP® 3Dx® or 4Dx® test kit**

	**2009**	**2010**	**2011**	**2012**	**2013**	**Cumulative**
SNAP tests utilized	1391	1346	1266	1127	1072	6202
SNAP *B. burgdorferi*-infection positive results	170	208	156	154	147	835
Old POS (+)	88	103	85	75	81	432
New POS (+)	82	105	71	79	66	403
Vaccine compliance status of tested dogs						
Fully vaccinated dogs tested	970	1053	933	833	762	4551
New POS (+)	11	11	10	11	3	46
Negative (−)	959	1042	923	822	759	4505
Incompletely vaccinated dogs tested (also includes non-vaccinated)	421	293	333	294	310	1651
Total new (+): (includes dogs with unknown vaccine status)*	71	94	61	68	63	357
New POS (+) among NON-vaccinated	56	76	55	55	49	291
New POS (+) among partially vaccinated	15	18	6	13	14	66
Negative (−)	350	199	272	226	247	1294

*Dogs with unknown vaccine status grouped (conservatively) with ‘Incompletely Vaccinated Group’: 2009 (12); 2010 (11), 2011 (0), 2012 (0), 2013 (1).

The summary of the cumulative test results (2009–2013) is displayed [Table 2]. The conditional probabilities for each vaccination condition are displayed within parentheses under the counts. Given that a dog is fully vaccinated, the probability of this dog testing positive in our sample is 1.01%. Similarly, given a dog is incompletely vaccinated, the probability of this dog testing positive in our sample is 21.62% [Table 2]. This appears to be a large practical difference in the percentage of dogs testing positive. However, because dogs are supposed to be tested annually for Lyme borreliosis, it is likely that the same dogs are being tested in different years. Therefore, in order to avoid artificially increasing the sample size and introducing dependence into the observations (since results from one dog may be related across different years), data were analyzed in yearly increments [21].

**Table 2:** **Cumulative summary of SNAP test results for** ***Borrelia burgdorferi*** **-specific antibodies in completely and incompletely vaccinated dogs (2009–2013)**

	**Positive**	**Negative**	**Total**
Completely vaccinated*	46	4505	4551
	(1.01%)	(98.99%)	
Incompletely vaccinated*	357	1294	1651
	(21.62%)	(78.38%)	
Total	403	5799	6202

*See working definition of completely and incompletely vaccinated dogs in Methods section.

### Statistical analysis

The summary of test results for each year beginning 2009 through 2013 is displayed [Table 3]. For 2009, the Chi-squared test statistic is 130.95 with a corresponding *p*-value less than 0.0001. Briefly this means that if there is truly no relationship between the SNAP test results for fully and incompletely vaccinated groups, the probability of collecting a sample with a relationship as strong as the one in our sample is less than 0.0001 [21]. For 2010 – 2013, the Chi-squared test statistics were 307.01, 137.89, 158.56, and 151.47, respectively with a corresponding *p*-values less than 0.0001 for each statistic [21].

**Table 3:** **The profile of a new-positive SNAP test result for** ***Borrelia burgdorferi*** **infection, including preventative fraction and risk ratio assessment among incompletely and completely vaccinated dogs**

	**2009**	**2010**	**2011**	**2012**	**2013**
Percent (+) of all tests run	12.22%	15.45%	12.30%	13.70%	13.70%
NEW (+) as % of all tests run	5.90%	7.86%	5.60%	7.01%	6.10%
Profile of a NEW (+) out of ALL new (+)’s					
Incomplete-vaccination	86.59% (71/82)	89.52% (94/105)	85.92% (61/71)	86.08% (68/79)	95.45% (63/66)
Full-vaccination	13.41% (11/82)	10.48% (11/105)	14.08% (10/71)	13.92% (11/79)	4.55% (3/66)
Infection rate (IR) based on vaccine status*:					
ALL incomplete-vaccination	16.86% (71/421)	32.08% (94/293)	18.32% (61/333)	23.13% (68/294)	20.32% (63/310)
Complete-vaccination	1.13% (11/970)	1.04% (11/1053)	1.07% (10/933)	1.32% (11/833)	0.39% (3/762)
Negative infection rate based on vaccine status					
ALL incomplete-vaccination	83.14% (350/421)	67.92% (199/293)	81.68% (272/333)	76.87% (226/294)	79.68% (247/310)
Complete-vaccination	98.87% (959/970)	98.96% (1042/1053)	98.93% (923/933)	98.68% (822/833)	99.61% (759/762)
Chi-square test statistic	130.95	307.01	137.89	158.56	151.47
P-value	<0.0001	<0.0001	<0.0001	<0.0001	<0.0001
Preventative fraction (PF) against infection**	93.29%	96.76%	94.16%	94.29%	98.08%
Risk ratio (RR)***	14.92	30.85	17.12	17.52	52.10

*Infection Rate = No. positive in vaccine status category/all dogs tested in that category. NOTE: All dogs other than those testing New (+) had (−) tests.

**PF = [(Incidence of Event in Untreated – Incidence of Event in Treated)/Incidence of Event in Untreated] × 100.

***Risk Ratio = % Infected when incompletely vaccinated/% infected when completely vaccinated.

The *Risk Ratio (RR)* describing the probability of *Borrelia burgdorferi* infection (as determined by SNAP) in incompletely vaccinated dogs compared to ‘fully’ vaccinated dogs between 2009 and 2013 averaged 21.4 (range 14.8-52.1) [Table 3]. The *Preventative Fractions (PF)* for prevention of a positive test result for each year beginning in 2009 through 2013 were 93.29, 96.76, 94.16, 94.29, 98.08, respectively [Table 3].

## Discussion

Four different canine Lyme borreliosis vaccines represented by six distinct brand names have been licensed by the USDA from 1992–2009. Immunizing against canine Lyme borreliosis can be achieved by delivering *either* a whole-cell bacterial lysate (bacterin) or a recombinant subunit vaccine containing Outer-surface protein A (OspA). In the current study, the recombinant OspA vaccine was chosen for its reported efficacy and safety profile and was recommended by the veterinary hospital as a ‘CORE’ vaccine for all dogs in 2009–2013. In each year analyzed, the risk of a dog testing seropositive for infection with *Borrelia burgdorferi* was greatly influenced by vaccine status; on average, over the 5-year study period just 1.01% (range of 0.39 - 1.32%) of fully vaccinated dogs exhibited evidence of a newly acquired infection while 21.62% (range 16.8 – 32.08%) of incompletely vaccinated dogs experienced a new infection [Table 3]. Using the modified frequency vaccination protocol wherein the scheduled first yearly booster was administered 6-months early, no adverse events were reported. Using Risk Ratio calculations, incompletely vaccinated dogs were between 14 and 52 times *more* likely to become seropositive for *B. burgdorferi* infection compared to their ‘fully’ vaccinated cohorts [Table 3]. In terms of a dog becoming newly positive for *B. burgdorferi*-specific antibodies (an indirect measure of infection), the Preventative Fraction when using the rOspA vaccine ranged from 93.29% in 2009 and 98.08% in 2013 [Table 3]. One limitation of this analysis is that the effect of tick preventative use (acaracides) between fully and incompletely vaccinated dogs was not determined. One could hypothesize that those dogs most likely to complete their vaccine series and remain vaccine protocol-compliant might be dogs with owners who are more likely to purchase tick control. Even if both groups purchased tick preventative products, it could be hypothesized that the fully compliant owners purchased, on average, more doses throughout the year. While additional analysis of the database may be useful to detect whether the variable of tick control played a role in Lyme-seropositive rates, the vast majority (>80%) of all canine patients within the hospital database receive less than 9 months of tick protection each year, regardless of their vaccine status. Given that infected *Ixodes scapularis* can transmit *Borrelia burgdorferi* whenever they attach to a suitable host, regardless of season, the influence of tick control (or lack thereof) between well vaccinated and incompletely vaccinated may be minimal. Beyond tick control, perhaps an analysis of newly positive SNAP tests by owner zip codes would reveal that *B. burgdorferi* infection pressure is greater in certain areas leading to new infections prior to completion of the recommended vaccine protocol. Lastly, while the Preventative Fraction figures related to seropositivity and vaccination status are extremely compelling, they are not substitutes for data derived from prospective, randomized, placebo-controlled studies. To be clear, the data from the study described here were not randomly collected, so it is not a comprehensive representation of the whole population.

## Conclusions

In this analysis, the practice owner’s unique interest in data-mining the hospital’s medical-record database to assess the rate of new *B. burgdorferi*-positive test results over a 5 year period is remarkable for its thoroughness and its findings. Furthermore, this analysis compliments and builds upon a previously published 21-month, 1,220 dog clinical observation study that showed that previously-negative, well vaccinated dogs rarely test positive for the presence of C6 antibodies specific for infection with *B. burgdorferi* [14]. In conclusion, for all the years from 2009 to 2013, the vaccination condition (e.g. ‘fully’ vaccinated versus not) and SNAP test results are not independent. We can therefore conclude that there is a strong, statistically significant relationship between compliance with this hospital’s immunization protocol using the canine recombinant OspA vaccine and the markedly low incidence rate of new cases of *B. burgdorferi*-positive test results (SNAP) in dogs [21].

## Abbreviations

SNAP: Brand name of IDEXX lyme antibody test kit
rOspA: Recombinant outer surface protein A
PF: Preventive fraction
RR: Risk ratio
*Bb*: *Borrelia burgdorferi*
